# Postural Orthostatic Tachycardia Syndrome Misdiagnosed as Anxiety: A Case Report with a Review of Therapy and Pathophysiology

**DOI:** 10.7759/cureus.10881

**Published:** 2020-10-10

**Authors:** Hassan Kesserwani

**Affiliations:** 1 Neurology, Flowers Medical Group, Dothan, USA

**Keywords:** autonomic disturbance

## Abstract

Dizziness can be protean with multiple phenotypes. One common phenotype in the young population is postural orthostatic tachycardia syndrome (POTS). POTS has a unique cardiovascular signature with a fascinating range of etiologies and pharmacodynamic substrates. This condition can pass undiagnosed for many years and is often mistaken as an anxiety disorder due to some of its hyperadrenergic manifestations. We present one such case and then flesh out the treatment strategies, both conservative and pharmacologic. We finally describe the various underlying pathophysiologic mechanisms of POTS and its sub-types and outline the various aberrant cardiovascular reflexes. We also describe the power spectra of the heart rate variability frequency bands and their underlying physiologic basis.

## Introduction

Postural orthostatic tachycardia syndrome (POTS) is an autonomic disorder defined by a heart rate rise of 30 beats/min or more within 10 minutes of standing or head-up tilt, in the absence of orthostatic hypotension. In children, the threshold increase in heart rate is 40 beats per minute. The standing heart rate may exceed 120 beats/min or higher [1].

The epidemiology of POTS is characterized by sexual dimorphism with a preponderance of women, 89% of cases. It is predominantly a disease of youth, typically occurring during adolescence and below the age of 25. The incidence rate for women aged 10-54 years is 17.9 per 100,000 person-years [2].

Symptoms of POTS fall into three major categories; those attributable to orthostatic intolerance and tachycardia such as lightheadedness, faintness, chest pain, and palpitations, which improve with supine posture. Cerebral symptoms include mental fog, non-specific headaches, migraine, and fatigue. The third group of symptoms is protean and includes myalgia akin to fibromyalgia, abdominal discomfort, urinary frequency and urgency, and insomnia. One-third of patients develop frank neurocardiogenic syncope and half of the patients describe an antecedent viral syndrome [3]. Careful patient history can often lead to the diagnosis of POTS, which can then be confirmed by provocative testing such as a head-up tilt table test.

Physiologically, standing leads to gravity-induced displacement of vascular blood from the upper to the lower half of the body. This involves the displacement of about 500 milliliters (ml) of blood. Without compensatory changes, systemic blood pressure may drop under the influence of gravity. However, the lack of influx of blood into the right atrium leads to three events. First, the atrial and aortic wall baroreceptors are offloaded, leading to reduced parasympathetic outflow and a rise in heart rate. Second, there is a burst of sympathetic activity leading to an increase in systemic vascular resistance and hence systemic blood pressure. These two mechanisms prevent a collapse in systemic blood pressure upon assuming the upright posture. The third event involves postural muscular contraction, involving mainly the calf muscles, which squeezes the blood vertically into the inferior vena cavae and henceforth further upstream eventually back to the right atrium. The venous circulation is also sympathetically contracted by alpha-adrenergic receptors [4].

This system of feedback loops can go aberrant in POTS. If the burst of sympathetic activity is excessive, there will be a hyperadrenergic response with orthostatic tachycardia. This is the rationale for the use of low-dose beta-blockers in POTS. If the lower extremity venous alpha-adrenergic receptors are down-regulated or dysfunctional, vascular efflux of blood from the lower extremities is diminished and venous return to the right atrium diminishes, leading to a drop in systemic blood pressure. This is the rationale for using midodrine, an alpha-adrenergic receptor agonist. A small subgroup of POTS patients has a depressed plasma aldosterone-to-renin ratio. This is paradoxical, as a low volume state should lead to a rise in renin and, subsequently, a rise in aldosterone levels. However, this so-called "low-flow" group leads to reduced renal sodium retention and plasma volume expansion. This is the rationale for using the mineralocorticoid fludrocortisone as pharmacotherapy. Finally, a small subset of patients has an elevated level of angiotensin II without concomitant hypertension. It is thought that this group of patients is insensitive to the vasoconstrictive effects of angiotensin II [5]. A more detailed account of pathophysiology and therapy will be outlined in the Discussion section.

In summary, POTS can be subdivided into the neuropathic POTS or hyperadrenergic POTS sub-groups. Neuropathic POTS is usually due to a partial autonomic neuropathy leading to impaired sympathetically-mediated vasoconstriction of the lower limbs, leading to venous pooling and impaired venous return. This is usually a post-viral phenomenon. Hyperadrenergic POTS is due to an excess sympathetic response. One must also rule out secondary causes of POTS due to circulatory insufficiency as seen with dehydration and physical de-conditioning after inanition and prolonged bed rest. Anxiety with its hyperadrenergic response can mimic POTS. Other rare conditions include pheochromocytoma, autoimmunity, as in voltage-gated potassium channel antibodies, glutamic-acid decarboxylase (GAD-65) autoimmunity, and cardiac conditions, such as arrhythmias or mitral valve prolapse, may also produce symptoms that overlap with POTS [3].

We present the case of a 19-year-old young woman who manifests with classic POTS symptoms and was misdiagnosed as anxiety for several years. This is quite a common presentation of dizziness in young people. This case highlights the misdiagnosis of POTS, which is relatively easily diagnosed with head-up tilt-table testing and its treatment, both conservative and pharmacologic. In the Discussion section, we briefly overview the common treatment strategies of POTS and then analyze the dynamics of the autonomic nervous system to help us better understand this autonomic insufficiency.

## Case presentation

We present the case of a 19-year-old young woman who has suffered from orthostatic intolerance since the age of 13. She develops lightheadedness upon standing up quickly with frequent spells of intermittent palpitations. On several occasions, she may develop near-syncopal episodes characterized by the dimming of vision, muffled sounds in her ears, and near collapse, with pallor and clamminess. Spells can be aborted by lying supine or by folding arms and bending the trunk. All her symptoms are aggravated by environmental heat and taking a hot shower. Many of her symptoms have been attributed to stress and anxiety with minimal benefit from anti-anxiety medications such as buspirone 5 milligrams (mg) twice daily and sertraline 25 mg daily.

Past medical history is negative for any illnesses. Family history is negative for autonomic insufficiency. She is attending nursing school.

On examination, blood pressure (BP) was 110/70 with a pulse of 98 beats per minute. The height is 5 foot and 7 inches, weight is 176 pounds with a body-mass-index (BMI) of 22.9. She was calm and answered questions directly. Cardiac auscultation revealed no murmurs. Her entire neurologic examination was normal except for mild jitteriness of the outstretched arms. Distal lower extremity discoloration was not noted.

Laboratory studies revealed a normal thyroid profile, with normal chemistries and the absence of anemia. A cardiac event monitor was normal. A 30-minute 70-degree tilt-table test was performed and the study was aborted at 10 minutes due to severe orthostatic intolerance and near syncope (Table 1).

**Table 1:** Tilt-table test results with body elevation at 70 degrees for 10 minutes, after which the study was aborted at 10 minutes secondary to intolerance Blood pressure (BP), heart rate (HR)

	supine posture	0 minutes	1 minute	5 minutes	10 minutes
BP	109/85	97/65	125/68	132/90	115/62
HR	61	106	117	122	124

With head-up tilting at 70 degrees, the patient developed a brisk tachycardia with a 63-point jump in heart rate. This was accompanied by palpitations, feelings of impending doom, lightheadedness, dimming of vision, and diaphoresis. The study was immediately aborted and the patient returned to the supine position. After five to 10 minutes, her symptoms resolved after adopting the supine position. A diagnosis of POTS was based upon the brisk and persistent rise in heart rate over 30 points within 10 minutes, leading to disabling orthostatic symptoms.

The patient was encouraged to enter an exercise program that included isometric leg exercises, such as cycling, and encouraged fluid intake, up to 10 cups a day (2.5 liters) and liberal salt intake, up to 10 grams a day, noting that a salt tablet contains 1 gram of sodium chloride. Due to the frequency of her symptoms and their debilitating nature, she was started on the mineralocorticoid, fludrocortisone, 0.1 mg daily. At the six-week follow-up, the patient reported almost complete resolution of her symptoms. A slow taper of buspirone was started with the aim of eventually weaning off sertraline too.

## Discussion

We review the commonly used agents used in the therapy of POTS and the Fast Fourier Transform analysis of heart rate variability and analyze the frequency bands of the sympathetic and parasympathetic system to help us better understand the dynamics of the autonomic nervous system. We also outline the various rarer etiologies of POTS.

Reassuring the patient that this condition is highly amenable to therapy is paramount, as many POTS patients go undiagnosed for years, and the condition can be debilitating, limiting social and physical activities. The treatment of POTS requires a two-pronged approach. Conservative measures, including physical counter-maneuvres, immediately during a POTS attack is useful to abort episodes. This can include crossing and uncrossing the legs and squeezing the muscles of the forearms in order to facilitate venous return. Lying supine or crossing the arms or legs and flexing the trunk can also help. Exercise to strengthen the leg muscles, such as cycling, rowing, and squatting exercises can help. Fluid intake up to 2.5 liters daily, which is equivalent to 10 standard cups a day and a total of 10 grams of sodium chloride daily, noting that one salt tablet contains one gram of sodium chloride, helps expand the plasma volume and improve venous return and the re-loading of the right atrium. Abdominal binders that increase intra-abdominal pressure and counter-pressure leg stockings can help improve venous return but these measures are not well-tolerated, especially in hot weather [6].

The most commonly used agents for POTS with mechanisms of action and practical tips are listed in Table 2 [7].

**Table 2:** Displaying the main pharmacologic agents used in treating the vast majority of POTS patients, with the mechanism of action and pragmatic notes Postural orthostatic tachycardia syndrome (POTS)

AGENT	CLASS OF AGENT	MECHANISM OF ACTION	NOTEWORTHY FEATURES
Fludrocortisone	Mineralocorticoid	Renal sodium retention with blood volume expansion and an increasing peripheral vascular resistance by sensitizing alpha-adrenergic receptors	Watch out for hypokalaemia
Midodrine	Vasopressor	Alfa-1 receptor agonist with venoconstriction and increasing venous return	Timing is important due to the short half-life. Watch out for supine hypertension
Pyridostigmine	Cholinesterase inhibitor	Increases synaptic autonomic ganglia acetylcholine thereby increasing vascular tone upon assuming erect posture.	Does not cause supine hypertension due to mechanism of action. Watch out for gastrointestinal distress
Propranolol	Beta 2-receptor blocker	Reduces tachycardia	Use low doses as higher doses can aggravate symptoms due to cardiac beta-2-receptor super-sensitivity

This is by no means an exhaustive list. Other agents, such as desmopressin tablets or nasal spray, droxidopa, and erythropoietin are very rarely prescribed. Cardiologists prefer more cardio-selective beta-blockers such as atenolol, metoprolol, or nebivolol. However, the vast majority of patients respond to conservative measures and the agents listed in Table 2.

Measurement of the power spectra over a 24-hour period using sophisticated mathematical techniques, such as Fast Fourier Transforms, reveals three frequency bands in the spectrum of heart rate variability (Table 3) [8]. 

**Table 3:** Power spectra analysis of heart rate variability reveals three frequency bands that reflect the autonomic activity of the nervous system Hertz (Hz)

	HIGH-FREQUENCY BAND	MID-FREQUENCY BAND	LOW-FREQUENCY BAND
FREQUENCY RANGE (Hz), not exact	0.2 - 0.4	0.04 - 0.15 (mean = 0.1)	0.02 - 0.07
PERIOD (seconds), not exact	2.5 - 7	7 - 25	25 - 300
ORIGIN	Parasympathetic - Respiratory	Parasympathetic - Sigh - Deep breath	Sympathetic activity / Cardiac sympathetic activity
PHYSIOLOGIC CORRELATE	Respiratory sinus arrhythmia	Baroreceptor activity	Thermoregulation / Renin-angiotensin system / Circadian rhythm
ONSET LATENCY(seconds)	Instantaneous	Instantaneous	Five-second delay; may reach steady state in 27 seconds
ABERRATION / PATHOLOGY	Cardiac disease / Aging / Anxiety disorders		Arrhythmic death / PTSD

**Figure 1:**
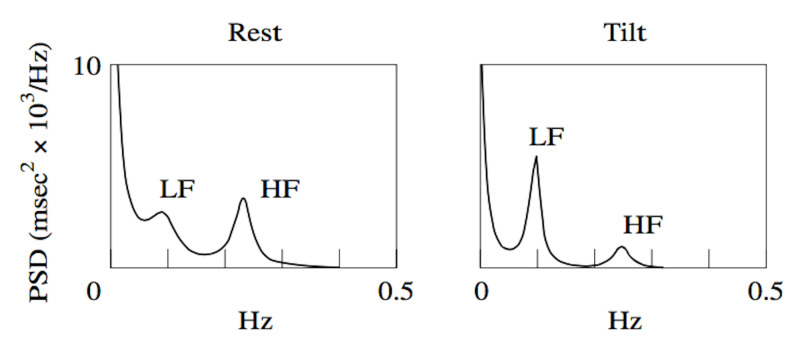
Shift in power spectra of low- and high-frequency bands from the supine to erect posture with head-up tilt Note the rise in LF peak and the fall in HF peak upon the assumption of erect posture. Power spectral density (PSD), millisecond (msec), Hertz (Hz), low frequency (LF), high frequency (HF)

In the supine position, the low-frequency band has a low peak implying low-level sympathetic activity and a high-peak high-frequency band implying vagal tone. On assuming the erect posture, the peakedness of the low- and high-frequency bands reverse with a rise in the low-frequency peak reflecting the adrenergic tone and activation of the renin-angiotensin system with the expansion of plasma volume. This is a long latency response with an onset latency of about five seconds and reaching a steady state in half a minute. In contrast, the high-frequency band, the parasympathetic response, is instantaneous and of a lower peak, paralleling the withdrawal of vagal tone with the assumption of the erect posture (Figure 1) [9].

In patients with POTS, head-up tilt table testing shows a higher low-frequency/high-frequency ratio implying a higher sympathetic tone upon assumption of the upright posture [10].

In neuropathic POTS, the spillover of norepinephrine into the venous circulation, a surrogate marker for the influx of norepinephrine into the circulation, is reduced. The trigger for norepinephrine release was induced by the cold pressor test, which is a painful stimulus leading to sympathetic activation, by the hypotensive effects of sodium nitroprusside infusion, which leads to the reflexive activation of baroreceptor-mediated sympathetic activation, and lastly by tyramine infusion, which depletes synaptic norepinephrine. All three methods led to a blunted surge of norepinephrine supporting the contention that there is restricted sympathetic denervation of the lower extremities with loss of sympathetic tone [11].

Hyperadrenergic POTS is associated with very high erect norepinephrine levels due to sympathetic overactivity or a hyperadrenergic state [12]. Strictly speaking, hypovolemia should be excluded. Pheochromocytomas usually lead to much higher norepinephrine levels, in association with metanephrines. A rare genetic mutation of the norepinephrine transporter protein leads to reduced synaptic uptake of norepinephrine and excess sympathetic stimulation that mimics POTS [13]. A remarkable condition associated with orthostatic tachycardia and episodic flushing is due to mast cell activation. This can be diagnosed by elevated urine methylhistamine. Aggravation of flushing reactions by beta-blockers due to mast cell degranulation and triggers such as sexual activity, the menses, and eating are diagnostic clues. Gastrointestinal distress is also a frequent complaint. Centrally acting agents, such as the alfa-2 agonist, clonidine, may help by reducing sympathetic outflow. Serotonin-norepinephrine reuptake inhibitors such as duloxetine or pure norepinephrine transporter inhibitors, such as atomoxetine, may also help [14].

Despite the prominence of cerebral symptoms, such as mental fogginess and neurasthenia in some patients, transcranial Doppler measurements and the computation of cerebral vascular resistance by the insonation of the middle cerebral arteries has revealed normal cerebral autoregulation in POTS [15].

Yu et al. found autoantibodies to angiotensin II in 12 out of 17 POTS patients. No autoantibodies were found in a control group of normal patients and patients with syncope. Those that did not harbor angiotensin II antibodies demonstrated alpha 1-adrenergic receptor antibodies [16]. Gunning III et al. demonstrated antibodies to alpha-1 adrenergic receptors in 89% and M4 anti-acetylcholine receptor antibodies in 53% of a cohort of 55 patients [17]. These studies support an autoimmune basis in a substantial number of POTS patients, opening up a window for potential immunotherapy in refractory POTS patients. Weinstock et al. report the successful treatment of POTS associated with mast cell activation syndrome with intravenous immunoglobulin [18].

## Conclusions

POTS is a remarkably common condition in the young population, which frequently goes undiagnosed and misdiagnosed. Our case is a classic presentation that was misdiagnosed as anxiety for several years. This is an important diagnosis to make, as POTS can be debilitating and is very amenable to therapeutic interventions, both conservative and pharmacologic. In this article, we highlight the cardinal manifestations of POTS, its clinical features, sub-types, and pathophysiology. We also outline the fascinating range of diseases that can lead to this syndrome. We place special emphasis on the aberrations of the autonomic reflexes, in particular, the underlying pharmacodynamic changes and the power spectra of the frequency bands of heart rate variability that reflect the activity of the sympathetic and parasympathetic arms of the autonomic nervous system.
